# Supplementary material for: Co-Expression Network Analysis Unveiled lncRNA-mRNA Links Correlated to Epidermal Growth Factor Receptor-Tyrosine Kinase Inhibitor Resistance and/or Intermediate Epithelial-to-Mesenchymal Transition Phenotypes in a Human Non-Small Cell Lung Cancer Cellular Model System
Source: Int J Mol Sci. 2024 Mar 29;25(7):3863. doi: 10.3390/ijms25073863 (PMC11011530; doi:10.3390/ijms25073863)
Supplement: Supplementary file 1 [file ijms-25-03863-s001.zip › Figure S1_fustaino et al.pdf]

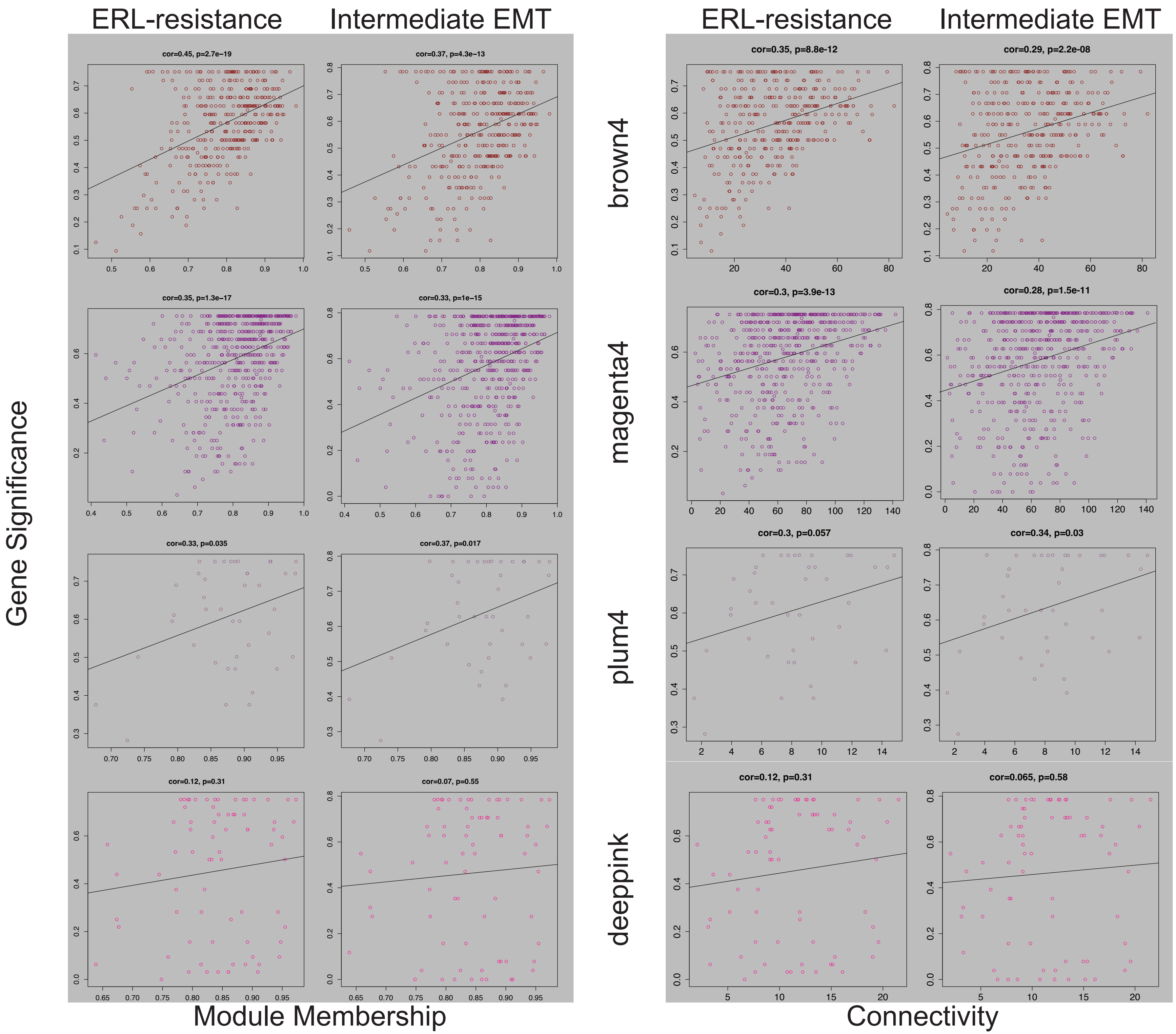

Figure S1. Correlation between WGCNA parameters. Scatterplots of gene significance for ERL-resistance and EMT intermediate phenotypes versus module membership and connectivity of selected modules. In brown4, magenta4, and plum4, gene significance for ERL-resistance and/or EMT intermediate phenotype exhibit good significant correlations (ρs > 0.3, p-value < 0.05) implying that hub genes of the module also tend to be correlated with the biological phenotypes of interest. Conversely, the module membership and connectivity parameters of the deeppink module members don't show any significant correlation with either ERL-resistance or EMT intermediate phenotypes. ERL = erlotinib; EMT = epithelial-mesenchymal transition.
