# Supplementary material for: Co-Expression Network Analysis Unveiled lncRNA-mRNA Links Correlated to Epidermal Growth Factor Receptor-Tyrosine Kinase Inhibitor Resistance and/or Intermediate Epithelial-to-Mesenchymal Transition Phenotypes in a Human Non-Small Cell Lung Cancer Cellular Model System
Source: Int J Mol Sci. 2024 Mar 29;25(7):3863. doi: 10.3390/ijms25073863 (PMC11011530; doi:10.3390/ijms25073863)
Supplement: Supplementary file 1 [file ijms-25-03863-s001.zip › Figure S3_Fustaino et al.pdf]

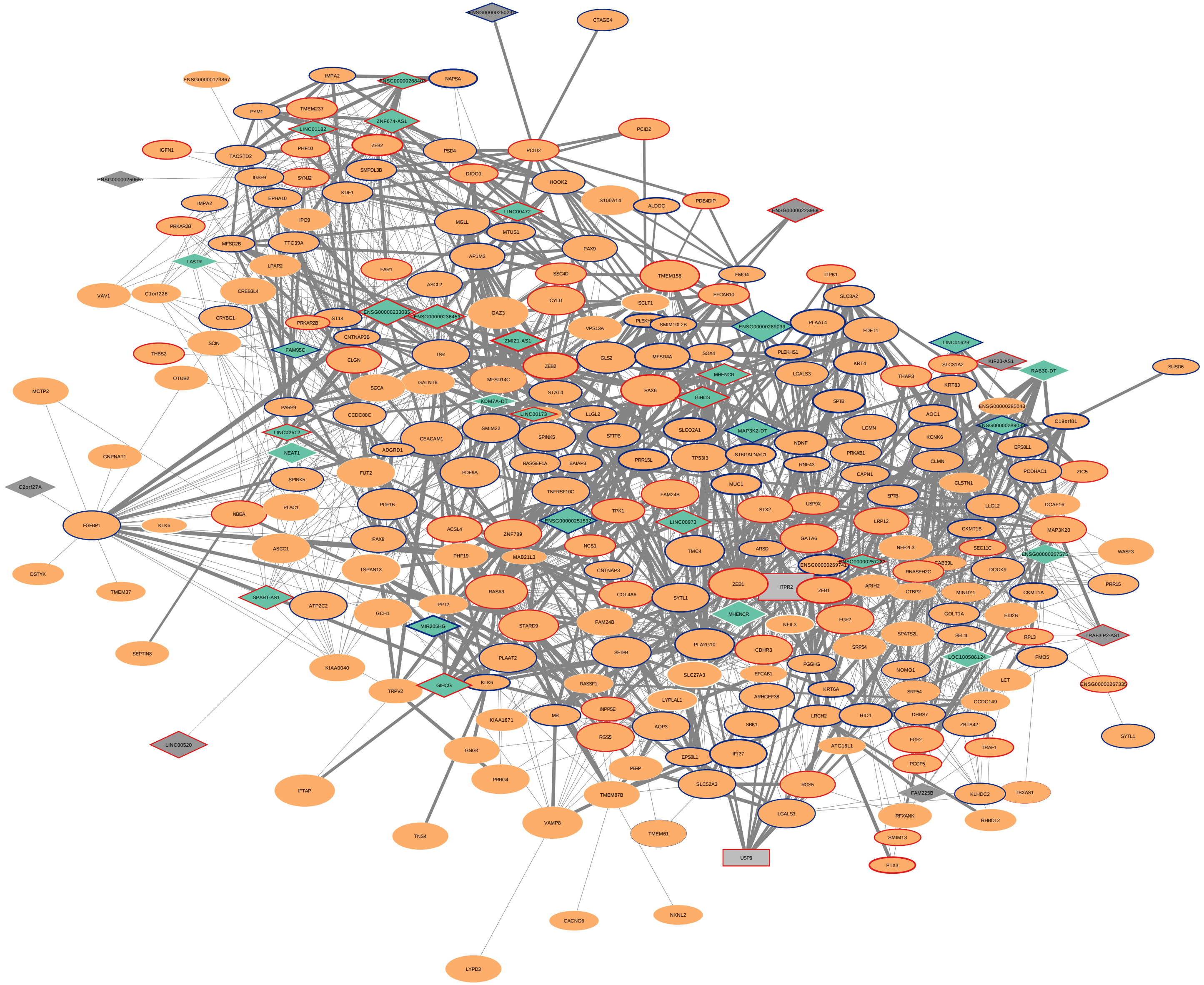

Figure S3. Network of the magenta4 module. lncRNAs (diamonds) and mRNAs (ellipses) at probe-level are shown in an organic layout. Nodes are connected by edges whose sizes are proportional to their WGCNA weights: the higher, the thicker. Node size is proportional to the intramodular connectivity measure (kWhithinScaled) and node border color and width indicate the fold-change between Erl-resistant/intermediate EMT, and Erl-sensitive/epithelial NSCLC cell lines: red = upregulation (|fold-change| > 1.5); blue = down-regulation (|fold-change| < 0.5); white = no expression variation; thickness= the higher, the thicker. Nodes of selected lncRNAs are filled in green.
