# Supplementary material for: Co-Expression Network Analysis Unveiled lncRNA-mRNA Links Correlated to Epidermal Growth Factor Receptor-Tyrosine Kinase Inhibitor Resistance and/or Intermediate Epithelial-to-Mesenchymal Transition Phenotypes in a Human Non-Small Cell Lung Cancer Cellular Model System
Source: Int J Mol Sci. 2024 Mar 29;25(7):3863. doi: 10.3390/ijms25073863 (PMC11011530; doi:10.3390/ijms25073863)
Supplement: Supplementary file 1 [file ijms-25-03863-s001.zip › Supporting Information revised_file.pdf]

Supplemental Information file

Co-Expression Network Analysis Unveiled lncRNA-mRNA Links Correlated to Epidermal Growth Factor Receptor-Tyrosine Kinase Inhibitor Resistance and/or Intermediate Epithelial-to-Mesenchymal Transition Phenotypes in a Human Non-Small Cell Lung Cancer Cellular Model System

Valentina Fustaino[1]*, Giuliana Papoff[1], Francesca Ruberti[1†] and Giovina Ruberti[1†,]*

[1]Institute of Biochemistry and Cell Biology, National Research Council (IBBC-CNR), Campus Adriano Buzzati Traverso Via E. Ramarini 32, 00015 Monterotondo (Roma), Italy; giuliana.papoff@cnr.it, francesca.ruberti@cnr.it

[†] These authors contributed equally to this work
*Authors to whom correspondence should be addressed valentina.fustaino@gmail.com; giovina.ruberti@cnr.it

The document contains the following files:

Figure S1. Correlation between WGCNA parameters. Scatterplots of gene significance for ERL-resistance and EMT intermediate phenotypes versus module membership and connectivity of selected modules. The absolute values of both module membership and gene significance are shown. In *brown4*, *magenta4*, and *plum4*, gene significance for ERL-resistance and/or EMT intermediate phenotype exhibit good significant correlations ($\rho_s > 0.3$, p-value < 0.05) implying that hub genes of the module also tend to be correlated with the biological phenotypes of interest. Conversely, the module membership and connectivity parameters of the *deeppink* module members don't show any significant correlation with either ERL-resistance or EMT intermediate phenotypes. ERL = erlotinib; EMT = epithelial-mesenchymal transition.

Figure S2. Network of the brown4 module. lncRNAs (diamonds) and mRNAs (ellipses) at probe-level are shown in an organic layout. Nodes are connected by edges whose sizes are proportional to their WGCNA weights: the higher, the thicker. Node size is proportional to the intramodular connectivity measure (kWhithinScaled) and node border color and width indicate the fold-change between Erl-sensitive/epithelial and -resistant/hybrid_intermediate NSCLC cell lines: red = up-regulation (|fold-change| > 1.5); blue = down-regulation (|fold-change| < 0.5); white = no expression variation; thickness= the higher, the thicker. Nodes of selected lncRNAs are filled in green.

Figure S3. Network of the magenta4 module. lncRNAs (diamonds) and mRNAs (ellipses) at probe-level are shown in an organic layout. Nodes are connected by edges whose sizes are proportional to their WGCNA weights: the higher, the thicker. Node size is proportional to the intramodular connectivity measure (kWhithinScaled) and node border color and width indicate the fold-change between Erl-sensitive/epithelial and -resistant/hybrid_intermediate EMT NSCLC cell lines: red = up-regulation (|fold-change| > 1.5); blue = down-regulation (|fold-change| < 0.5); white = no expression variation; thickness= the higher, the thicker. Nodes of selected lncRNAs are filled in green.

Table S1.xls Gene expression data and WGCNA results of whole NSCLC cell line dataset.

Table S2.xls Phenotypical traits of cell lines.

Table S3.xls Gene expression data and WGCNA results of whole NSCLC cell line dataset of the *brown4* module.

Table S4.xls Gene expression data and WGCNA results of whole NSCLC cell line dataset of the *deeppink* module.

Table S5.xls Gene expression data and WGCNA results of whole NSCLC cell line dataset of the *magenta4* module.

Table S6.xls Gene expression data and WGCNA results of whole NSCLC cell line dataset of the *plum4* module.

Table S7.xls Results of DAVID functional enrichment analysis of *brown4* module. The last column indicates the protein coding genes connected to selected lncRNAs in the WGCNA network.

Table S8.xls Results of DAVID functional enrichment analysis of *magenta4 module.* The last column indicates the protein coding genes connected to selected lncRNAs in the WGCNA network.

Table S9.xls Results of DAVID functional enrichment analysis of *plum4* module. The last column indicates the protein coding genes connected to selected lncRNAs in the WGCNA network.

Table S10.xls Details of the top lncRNAs correlated to intermediate epithelial to mesenchymal transition (int-EMT) and/or erlotinib resistance (ERL-res).

Table S11.xls Sequences of oligonucleotide primers used in qPCR in Figure 4.
