# Supplementary material for: Co-Expression Network Analysis Unveiled lncRNA-mRNA Links Correlated to Epidermal Growth Factor Receptor-Tyrosine Kinase Inhibitor Resistance and/or Intermediate Epithelial-to-Mesenchymal Transition Phenotypes in a Human Non-Small Cell Lung Cancer Cellular Model System
Source: Int J Mol Sci. 2024 Mar 29;25(7):3863. doi: 10.3390/ijms25073863 (PMC11011530; doi:10.3390/ijms25073863)
Supplement: Supplementary file 1 [file ijms-25-03863-s001.zip › Table S11_Fustaino et al.pdf]

Table S11. Sequences of oligonucleotide primers used in qPCR in Figure 4

Name	Sequence (5’-3’)	
LINC-PINT-FW	GAACGAGGCAAGGAGCTAAA	**BROWN4**
LINC-PINT-RV	AGCAAGGCAGAGAAACTCCA	
LYPLAL1-DT-FW	ACTGACTGTTGCATTCATCTCTCT	
LYPLAL1-DT-RV	CAGACTTTGAATGGCTATGGCAC	
MIR100HG FW	ACCCCTCCTCAGGAATGTCT	
MIR100HG RV	GCAGAGGAGGTGTCTTCAGG	
GIHCG-FW	GCTTCTCTAGTTTTCTGCCTCCT	**MAGENTA4**
GIHCG-RV	ATTGTCTTCACATGCTGTGTCCA	
MIR205HG-FW	GGAGTGCAGTGGCTCAATCT	
MIR205HG-RV	TGGATTGCTTAAGCTCAGGA	
ZMIZ1-AS1-FW	ACTTTTCAGTGACTTCCACTGGT	
ZMIZ1-AS1-RV	AGCTCCCTGGTAGAATTCCCT	
SATB2-AS1-FW	GTCCGGTGTGGGTTTTTGTC	**PLUM4**
SATB2-AS1-RV	TTGTGCAGTAAAAAGCGTATGT	
RPL31-FW	CATCCATGGAGTGGGCTTCA	**Reference gene**
RPL31-RV	AGCTTTGTTGAGCCTGGTGT	
